# Miscellaneous Facts and Remarks

**Published:** 1799-09

**Authors:** 


### *Miſcellaneous Facts and Remarks.*

FROM a letter of Dr. L. Maclean, dated Sudbury, Auguſt 18th, we extract the following paſſages, relative to the uſe of the *fox-glove*, as connected with his paper, inſerted p. 113, and fol.

"As I perceive your ingenious correſpondent Dr. Mossman, appears deſirous of giving the fox-glove a fair trial in conſumption, I truſt I may be ſuffered to preſume on the experience I have had of this medicine in pulmonary affections, by aſſuring him he is not likely to be ſucceſsful while he continues to preſcribe it as he now does. In one caſe he directs gr. j. to be adminiſtered every two hours. Now, as this quantity of the powder of of the genuine leaves, prepared as directed in the abstract lately transmitted to you, would, in less than twenty-four hours, produce in most habits very powerful effects, I am induced to infer, that instead of the active fox-glove, his patients were using the inert powder of half-decayed leaves, a circumstance I have repeatedly experienced when I first began to prescribe it. This I am persuaded has been the case with many practitioners, and must frequently happen, without the strictest attention to the mode of preparing it.

"It is only by the most gradual exhibition of the medicine, and by keeping the habit for some weeks under its influence, that any permanent advantage is to be derived in the worst cases of phthisis; and gr. j. of the powder, or from fifteen to twenty-five drops of the tinctures of which I have given formulas, taken morning, noon, and night, will in general be found sufficient to do this.

"In the case of a young woman now under my care, of confirmed hereditary consumption, twenty-nine drops of the tincture have been taken, *ter in dies*, but she has been for some days so much under its dominion, that I judged it prudent yesterday to lessen the dose to twenty drops. The pulse has never been reduced lower than 120, but one day when it was 112 in a minute, although she has been taking it for upwards of a fortnight, and it was increased in the most gradual manner. No advantage has as yet been obvious, nor am I very sanguine. Pray have the goodness to add, after the formula I have given of the infusion, "a table-spoonful of which, taken three times a day, is a full dose for an adult to begin with."

---

The American continent in general, and the United States in particular, are very much engaged in ascertaining the real causes of their epidemic diseases, and the best means of preventing them. The Transatlantic Journals contain many ingenious and instructive papers on this subject; especially those relative to Dr. MITCHILL's theory of the *septic acid*, which at present appears to be by far the most prevalent. Although these subjects are of the first importance to the practitioner in warm climates, we are inclined to believe, that the articles we have selected, will be equally interesting and useful to all medical readers.

---

Dr. RUSH mentions in the fifth volume of his "*Medical Inquiries and Observations*," a striking illustration of the *good effects* of *depletion*, as a preparative in strangers, to encounter the dangers of a baneful climate, in a communication from Dr. BORLAND, one of the physicians of the British military hospitals in the West-Indies.

"In the beginning of August, 1797, 109 Dutch artillery-men arrived at Port-au-Prince, in the Bangalore transport. The florid appearance of the men, their heavy, cumbersome cloathing, and the season of the year, seemed all unfavourable omens of the melancholy fate, we presumed, awaited them. It was, however, thought a favourable opportunity by Dr. JACKSON and myself, to try what could be done in warding off the fever. It was accordingly suggested to M. CONTURIER, the chief surgeon of the foreign troops, and the surgeon of the regiment, that the whole detachment should be blooded freely, and that on the subsequent morning a dose of physic should be administered to every man. This was implicitly complied with in a day or two after; and at this moment in which I write, although a period of four months has elapsed, only two of that detachment have died, one of whom was in a dangerous state when he landed. A

success unparalleled during the war in St. Domingo. It is true, several have been attacked with the disease, but in these the symptoms were less violent, and readily subsided by the early use of the lancet. The crew of the Bangalore, on her arrival at Port-au-Prince, consisted of twenty-eight men. With them no preventive plan was followed: in a very few weeks eight died; and at present, of the original number only fourteen remain.

"It is curious to notice, in connection with the above narrative, a fact quoted by Dr. Rush, from Dr. M'Kitterick, that the heat of the body in strangers, newly arrived in the West-Indies, has been found to be between three and four degrees above that of the temperature of the natives. How far such depletion, as that practised in the case of the Dutch artillery at St. Domingo, might be useful at the beginning of an epidemic season in the United States, may be left to the decision of future experience."—*Medical Repository*, Vol. II. p. 190.

---

Dr. Williamson, of New-York, after having stated several instances, proving the *ill effects of blood-letting in putrid bilious fevers*, and *pneumonia typhoides*, as they appear in North-Carolina; and after observing that he did not hear, during a whole winter season, of a single case of a patient recovering who had been freely bled, concludes with the following judicious remarks:

"As the patients," says Dr. Williamson, "who suff[illegible]r by the complaint are commonly men, not often women, and as men expose themselv[illegible] much more imprudently than women to the cold and to the rain, there is reason to believe, that a checked perspiration is the proximate cause of the complaint. Any fever, thus induced, where the fluids are dissolved, and in such a state of the atmosphere as has been mentioned, must soon be expected to put on a dangerous appearance. I have known a man, thus prepared by intermittents, in the season and country mentioned, bring on by dancing, what was called a pleurisy in his head, and die in forty-eight hours.

"I was assured by Dr. Sawyer, a physician in Pasquetank, about thirty miles from Edenton, that he was called to many patients in the winter of 1792. They complained of a pain in the side, and a high fever. He seldom waited, as he declared, even for a remission: he gave the bark in substance, and his patients recovered. Having business in Pasquetank, I inquired concerning the general progress of the fever during that sickly season. I was assured by a gentleman there, whom Dr. Sawyer had attended, that having a high fever and considerable pain, he took the bark in substance, and thought that his fever moderated by every dose. He said that one of his slaves had been treated in the same manner, with a similar effect. He added, that another physician in the same vicinity, a man of talents, and well-educated, adhering to the bleeding system, while the pain and fever remained, had lost the most of his patients.

"Whatever success may have attended the practice mentioned, I think the indiscriminate and immediate use of the bark must be exceptionable and hazardous. I am nevertheless persuaded, by all the observations I have made on this subject, that the lancet should seldom be used in the brumal fevers, which often appear in the low country in Carolina; but it is a remedy commonly at hand—it promises immediate relief to a person in pain; these circumstances appear to have kept it too long in use. *Ibid.* pp. 156—159.

---

Mr. Daniel D. Walters, apothecary, in New-York, relates of himself the following case of the *dysenteric effects of nitrous acid* taken into the the ftomach. He had exercifed himfelf confiderably on a day, near the end of April, 1798, and had become thirfty; entering his fhop in this fituation, he took a large bottle which he fuppofed to contain water, applied it to his mouth, and drank heartily; immediately after fwallowing the laft mouthful, he was fenfible of a fourifh, difagreeable tafte, and on inquiry found that his apprentice, inftead of putting the water for drinking into the ufual bottle, had poured it into another bottle of the fame material, fize, and capacity, ftanding befide it: neither of them being marked or lettered, and the bottle into which the water, by miftake, had been poured, was the one in which aqua-fortis had been kept, and not completely poured out. The liquid he fwallowed was therefore a diluted aqua-fortis. Within a few minutes a deadly and intolerable naufea came on, which, within a quarter of an hour, ended in vomiting. After this he felt better for fome time, when pain, griping, and flatus fucceeded, and about fifteen hours after taking the dofe, tenefmus and bloody ftools followed; thefe latter fymptoms were attended with head-ach and fever; the dyfentery lafted two days, and gradually went off, no medicine having been taken.

The brother of Mr. Walter, who drank of the fame acidulated water, was alfo exceffively incommoded by it, and placed in extreme danger. *Ibid.* pp. 337 and 338.

---

Dr. MITCHILL, of New-York, has lately addreffed a letter to Sir JOHN SINCLAIR, *on the affinities and relations of feptic* (nitric), *or peftilential fluids to other bodies*; intended as an additional article to the propofed Report of the Britifh Board of Agriculture, on the fubject of manures—from which we extract the following curious paffages:

"While I was engaged," fays Dr. Mitchill, "in inveftigating the manner in which peftilential fluids exerted their powers on the human body, and juft after I had arranged the materials of my letter to Mr. HAVENS, an occurrence of a kind novel to myfelf befel a man in the Infirmary of New York. Being then in attendance as a phyfician, I was requefted to take under my charge one of the patients in the furgeon's ward, who had for feveral days before been feized with an intermitting fever, and feverely handled by it. On inquiring into the particulars of his indifpofition, I found the complaint for which he had been admitted into the houfe was an ulcerated leg. This, for fome time, had been dreffed with the red precipitate (feptite of quickfilver), in fuch large quantities, that when at the cuftomary time the furgeon's affiftant renewed the dreffings, the bottom and fides of the fore were frequently covered with it, in its undecompofed ftate. Such a long-continued and plentiful application of the efcharotic feemed to have afforded feptic (nitric) acid enough, to be abforbed into the veffels, and apparently to produce confiderable effects on the conftitution. To this interpretation of the difeafe I called the attention of the byftanders, and the circumftance of the ulcer, with the febrile fymptoms, had fo much of the probability of caufe and effect, that we were generally of opinion, the feverifh commotion might have been ftirred up by the acid abforbed, after its difengagement from the mercury.

"Some precious remarks on the fixation of the feptous (nitrous) vapours of manures by earthy fubftances, are to be found in SCANDELLA's letter to ARDUINO *. The author's obfervations, which were made for the Venetian territories, apply with great force to New-York, whofe fouthern part is nearly

* See Outlines of the 15th Chapter of the General Report, &c. Addend. p. 12.

nearly the ſame parallel of latitude with the north of Italy. Scandella conſiders putrid diſeaſes in the ſouthern parts of Europe, if not originating from the ſeptic exhalations of dunghills and heaps of manure as their only cauſe, as certainly rendered more dangerous by them. He obſerves that they "may be looked upon as not far diſſimilar from marſh-miaſmata, and may "afford a fomes to periodical fevers." And in manufacturing manure in hot climates, he gives particular cautions againſt the putrid exhalations which it emits.

"The people of Great Britain may imagine themſelves ſecure from the noxious effect of theſe peſtilential ſteams; but I have a confidence, if the matter was properly inquired into, they would find not only their jail, and ſhip, and hoſpital fevers originating from this effluvium, but that among their poor peaſantry and manufacturers, though it is not concentrated enough to cauſe the high-wrought forms of febrile ailments in America, yet their typhus, in all its gradations, is fairly to be aſcribed to the more feeble and gradual operation of theſe ſeptic (nitric) vapours.

"This kind of compound ſeems alſo to have been *almoſt* expreſſed by Darwin (*Zoonomia.* vol. 1. ſect. xxviii. 2.), where the *hectic fever* ſupervening, by admitting air to an ulcerated ſurface, on opening an abſceſs, is aſcribed by that elegant and original philoſopher, to the *azotic*, rather than the oxygenous portion of the atmoſphere; though he appears ſince to have changed it to the *oxygen*, in which he has probably not bettered his judgment; the preferable opinion being, that they are *both* inſtrumental in ſtirring up febrile commotion in the ſyſtem, by forming a ſeptic fluid, producing, with an allowance for peculiarity on attendant circumſtances, an effect analogous to the patient whoſe caſe I related in the beginning of this letter.

"It ſurpriſes me, in looking into Schmeisser's edition of Von Uslar's Obſervations on Plants, that ſo little is ſaid concerning the operation of the *ſeptic* principle (azote) on the vegetable economy; but ſepton (azote), ſo long overlooked, will probably attract more attention for the future." *Ibid.* pp. 345—353.

---

Dr. Nooth, Superintendant-General of the hoſpitals in Britiſh America, in a letter to Dr. Mitchill, dated Quebec, Jan. 24, 1799, gives the following opinion reſpecting the *treatment of dyſenteries, and other autumnal diſeaſes*:—"I can aſſure you," ſays he, "I am almoſt convinced of the truth of your doctrine with regard to azote, or ſepton, as the cauſe of thoſe epidemic diſeaſes which generally make their appearance toward the end of ſummer, in warm countries. There are ſome obſervations which I have made in my own practice, that lead me to ſuppoſe, there may be *ſomething of an acid nature*, that may act as an exciting cauſe in the dyſentery and yellow fever, and indeed in many other diſeaſes that ariſe from heat and other circumſtances, in the latter months of the warm ſeaſon.

"Having unfortunately ſeen, in the courſe of my practice, a great number of dyſenteric caſes, and having experienced the inefficacy, in general, of the uſual mode of practice, I was induced to try the effects of the ſeveral purgatives now in uſe, with the view of aſcertaining how far any one was preferable to the others, in the treatment of dyſenteric patients. Experience ſoon taught me, that the *tartarum ſolubile* (neutralized tartarit of potaſs) was the moſt ſalutary in its effects; and of courſe, I have always, ſince that diſcovery, had recourſe to it in dyſenteries and other autumnal diſeaſes; and, I can aſſure you, with the greateſt ſucceſs, both in children and adults. The component parts of that neutral (acid of tartar and potaſs), and and the advantages attending its use in the cases above alluded to, seem to confirm your doctrine, and induce me to believe your theory is better founded than the world will, perhaps, at first allow. *Ibid.* p. 437.

---

Under this head of our Journal we wish, at present, to revive in the memory of our readers, the *alexipharmic powers of pure ammonia*, or caustic vegetable alkali, in the cure of persons bitten by snakes in hot climates. The dose is sixty drops in a sufficient quantity of water, taken internally, till the symptoms abate. The wound ought also to be washed with the same solution. This treatment has always been attended with success, when the patient was able to swallow the medicine.

---

Dr. Richter, of Göttingen, has lately described a particular species of dropsy, in a very interesting and useful work, entitled "Medical and Chirurgical Observations," page 268, of the original German; a faithful English translation of which appeared in 1794, by Dr. Spens, of Edinburgh. As this work is little known among our medical friends in this country, we presume to repeat, that Dr. Richter derives the *hydrops vagus*, or the vagrant dropsy, from a spasmodic state of the lymphatic system, arising from any particular stimulus. This may be either of a gastric, bilious, rheumatic, arthritic, venereal, or any other kind, so that it may affect any part, or organ of the body, exclusively. This theory appears to be perfectly consonant to nature, and to explain many pathological and therapeutical difficulties, especially to those who are accustomed to treat all dropsical complaints indiscriminately with diuretic remedies.

Prof. Hecker, of Erfut, is so much convinced of the truth of this hypothesis, that he has illustrated it with a remarkable instance communicated to the Editors of the "*Journal der Erfindungen*," who have given it a place in their tenth Number. As our limits do not admit of relating this case at full length, we shall only remark that the learned Professor explains the cause of that disease in his patient, from the irritable and spasmodic state of the whole system; from the peculiar stimulus which first produced gastric symptoms, preternatural sensibility of the stomach, pain in the side, fever, and afterwards the hydrops vagus. The good effects of antispasmodic remedies likewise appear to confirm the ætiology of Dr. Richter, in a great variety of dropsical cases: nor does either of these learned writers maintain, that spasms are the *only* cause of *all* dropsies.

Another important fact tending to corroborate this theory, we find in the last volume of "New Observations and Facts, 1795," by the venerable Theden, Surgeon-General to the Prussian armies. He recommends, from long experience, in dropsical cases after quartan agues, the use of the belladonna, the leaves of which afford the best and most effectual remedy. It is scarcely possible to conceive any other effect from this medicine, than that of removing or suppressing the irritable state of the nervous system, of which dropsy frequently is the consequence.

In the opinion of Prof. Hecker, even the *digitalis* (which is perhaps too generally prescribed in dropsy) appears to possess no specifically diuretic virtues. This medicine obviously diminishes irritability, so that *the pulse becomes slower*, while it moderates tensions and spasms. Its resolvent and diuretic powers, therefore, must be ascribed chiefly to those effects. Hence it will most probably effect a cure in spasmodic dropsy, but prove of no service in that arising from other causes: and thus we may account for the contradictory opinions which have hitherto prevailed respecting the efficacy of this medicine.

